# Hepatic eosinophilic granuloma without peripheral eosinophilia diagnosed by laparoscopic liver resection: a case report

**DOI:** 10.1097/RC9.0000000000001100

**Published:** 2026-10-22

**Authors:** Takao Tayama, Tomoyuki Abe, Hiroki Kitagawa, Tsuyoshi Kobayashi, Susumu Tazuma, Hideki Ohdan

**Affiliations:** a Department of Gastroenterological and Transplant Surgery, Graduate School of Biomedical and Health Sciences, Hiroshima University, Hiroshima, Japan; b Department of Infectious Disease, Hiroshima University, Hiroshima, Japan; c Department of Gastroenterology, Hiroshima Prefectural Hospital Organization Futabanosato Prefectural Hospital, Hiroshima, Japan

**Keywords:** case report, eosinophilic granuloma, laparoscopic hepatectomy, parasitic infection

## Abstract

**Introduction and importance:**

Eosinophilic granuloma is a histopathological condition characterized by eosinophil-rich granulomatous inflammation and is considered a manifestation of allergic reactions. Hepatic eosinophilic granuloma (HEG) is an extremely rare entity and is most commonly associated with parasitic infections. We report a case of HEG without peripheral eosinophilia or a confirmed parasitic infection, with a difficult preoperative diagnosis, treated by laparoscopic liver resection.

**Case presentation:**

A 67-year-old woman was referred for further evaluation of a liver tumor in segment 3 detected by computed tomography performed for unexplained abdominal pain. Tumor markers were not elevated, and although further imaging studies did not exclude hepatic malignancy, an inflammatory pseudotumor or intrahepatic cholangiocarcinoma was suspected. Laparoscopic sub-segmentectomy of segment 3, and lymph node dissection around the common hepatic artery, were performed for diagnostic and therapeutic purposes, respectively. Histopathological examination revealed a granuloma with marked eosinophilic infiltration and central coagulative necrosis. Tests for mycobacterial infection were negative, and neither parasitic organisms nor serological evidence of parasitic infection was identified.

**Clinical discussion:**

Preoperative diagnosis of HEG remains challenging because its radiological findings often resemble those of hepatic malignancies, particularly in the absence of peripheral eosinophilia or evidence of parasitic infection.

**Conclusion:**

HEG is most frequently associated with parasitic infections. However, precise preoperative diagnosis of nonparasitic HEG remains challenging. Laparoscopic resection enabled a definitive diagnosis. Therefore, HEG should be considered in the differential diagnosis of primary hepatic tumors, even in the absence of confirmed parasitic infections or peripheral eosinophilia. Minimally invasive surgery is a reasonable option from both therapeutic and diagnostic perspectives.

## Introduction

Eosinophilic granulomas (EGs) are pathognomonic findings associated with various underlying conditions. Hepatic eosinophilic granuloma (HEG) is extremely rare and usually caused by parasitic infections. The predominant hypothesis is that type I hypersensitivity reactions to parasitic antigens cause vasculitis and subsequent eosinophil aggregation, resulting in secondary HEG formation^[^1,2^]^. This phenomenon is often referred to as visceral larva migrans and is associated with various parasites such as *Toxocara* and *Capillaria species*, liver flukes, *Anisakis,* and *Schistosoma* species^[^3–10^]^.

HIGHLIGHTS

- Hepatic eosinophilic granuloma is an extremely rare benign hepatic lesion.
- Preoperative imaging findings may closely mimic a primary hepatic malignancy.
- Laparoscopic hepatectomy enabled a definitive diagnosis in this challenging case.

Despite the well-established association between HEG and parasitic infections, a definitive diagnosis remains challenging. The current gold standard often requires a histopathological examination of the resected tissue^[^1^]^. However, a considerable diagnostic hurdle is that the causative parasites are not consistently detectable using serological, histopathological, or immunochemical methods^[^1,4^]^. Consequently, HEG often presents a diagnostic dilemma, and the differential diagnosis commonly includes malignant conditions such as cholangiocarcinoma and liver metastases^[^1,5,11^]^. Radiological characteristics of HEG are similar to those of liver abscesses^[^9,12^]^. This ambiguity underscores the necessity for reliable and non-invasive diagnostic strategies to accurately identify HEG. Given these considerations, surgical resection of HEG is acceptable for diagnostic and therapeutic purposes^[^1^]^.

Herein, we report a case of successful surgical resection of nonparasitic HEG in which radiological findings mimicked intrahepatic cholangiocarcinoma (ICC).

This case report has been reported in line with the SCARE 2025 criteria.

## Case report

A 67-year-old woman was referred to our hospital for a thorough evaluation of a newly discovered liver mass. The patient experienced spontaneous resolution of acute abdominal pain a few days before visiting a local clinic. Contrast-enhanced computed tomography (CT) at the referring clinic revealed a 41 mm hypovascular mass in segment 3 (S3) of the liver, with its periphery showing progressive enhancement in the late phase (Fig. 1A and B). A 15 mm flattened, enlarged lymph node was observed around the common hepatic artery. Her medical history included an oophorectomy and hysterectomy for uterine fibroids. She took no regular medications and had no history of owning pets, traveling abroad, or allergies. Upon initial presentation, her vital signs were normal, and the laboratory results revealed a mild elevation in inflammatory markers and liver enzymes. Her white blood cell count was 5 680/μL, and no peripheral eosinophilia was observed (7.2%, 410/μL). Tumor marker levels were within normal ranges (Table 1). The lesion showed gradual peripheral enhancement on dynamic CT (Fig. 1). Ethoxybenzyl-enhanced magnetic resonance imaging revealed a hypointense tumor on T1-weighted images with slight peripheral hyperintensity on T2-weighted images. On dynamic contrast-enhanced imaging, the tumor showed faint reticular enhancement along the periphery with poor central enhancement. The surrounding liver parenchyma showed relative hyperenhancement during the arterial phase. Diffusion-weighted imaging revealed peripheral hyperintensity, and the hepatobiliary phase indicated minimal Primovist uptake in the tumor (Fig. 2). A positron emission tomography–CT scan exhibited increased fluorodeoxyglucose uptake, with a maximum standardized uptake value (SUVmax) of 10.3 in the main tumor and an SUVmax of 7.3 in a lymph node at Station 8a, with no evidence of distant metastasis. Preoperative differential diagnoses included inflammatory pseudotumors, ICC, and metastatic liver tumors. Although preoperative tumor biopsy was considered, resection was prioritized, as complete tumor resection was deemed feasible, enabling definitive diagnosis and treatment. A laparoscopic subsegmentectomy of S3 was performed. Intraoperatively, contrast-enhanced ultrasonography with perflubutane (Sonazoid) revealed a peripheral ring-like enhancement in the vascular phase and a corresponding perfusion defect in the Kupffer phase. Intraoperative frozen section analysis of the lymph nodes at Station 8a confirmed the absence of malignancy. The operative time was 280 minutes, with an estimated blood loss of 20 mL. The patient’s postoperative course was uneventful. The patient was discharged on the tenth postoperative day.

**Figure 1.**
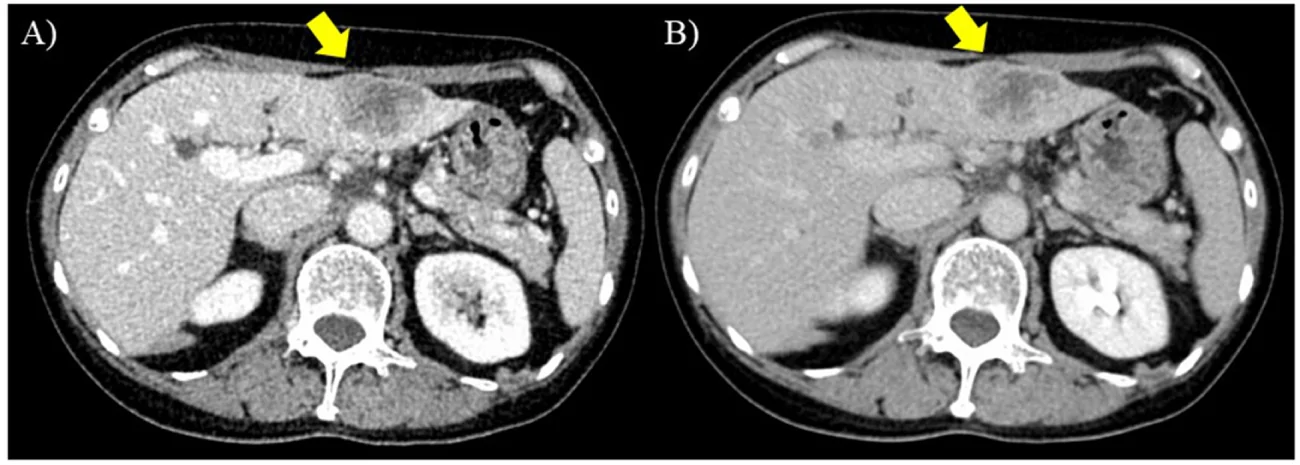
Contrast-enhanced abdominal CT: (A) arterial phase; (B) delayed phase. A 41 mm hypovascular mass on the surface of segment 3 of the liver shows progressive peripheral enhancement in the delayed phase.

**Figure 2.**
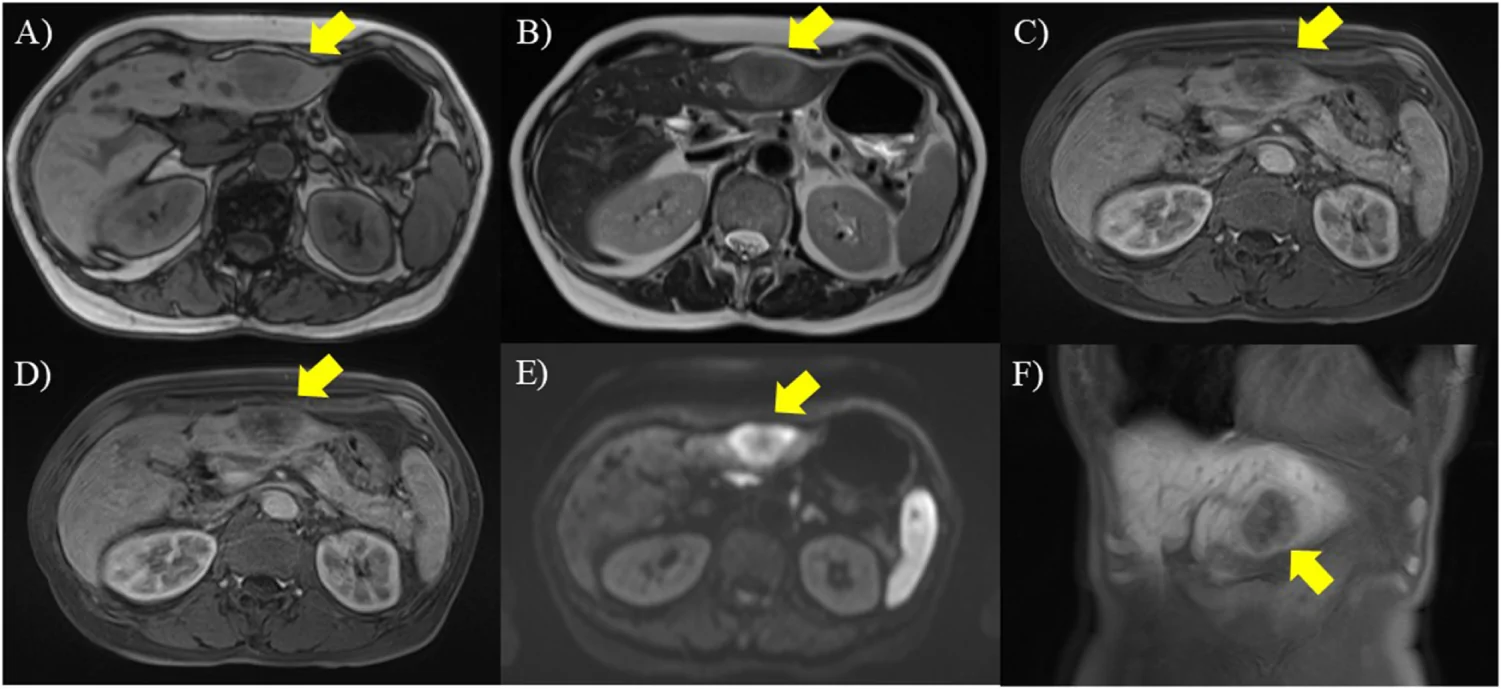
MRI findings: (A) T1-weighted image showing a hypointense lesion. (B) T2-weighted image: The peripheral portion of the lesion shows a slight hyperintensity. (C) Dynamic contrast-enhanced imaging: The tumor shows faint reticular enhancement, predominantly at the periphery, with poor enhancement in the central area. (D) Arterial phase: the lesion is relatively hyper-enhanced compared to the surrounding liver parenchyma. (E) Diffusion-weighted image: high signal intensity is observed at the periphery of the lesion. (F) Hepatobiliary phase (coronal): slight ethoxybenzyl uptake is observed at the tumor margin, possibly indicating delayed washout.

The pathological findings were as follows. Gross examination of the resected specimen revealed a well-demarcated, whitish-yellow nodular lesion (Fig. 3A). Microscopic pathological examination revealed a large granuloma with prominent eosinophilic infiltration (Fig. 3B). The central portion of the granuloma showed extensive coagulative necrosis (Fig. 3C). There was no evidence of bile duct stasis or vasculitis within the intrahepatic or small bile ducts. Eosinophil-specific staining, as well as staining for parasitic eggs and foreign bodies, were negative. Neither immunohistochemical staining nor screening for antiparasitic antibodies (SRL Inc., Tokyo, Japan) revealed any significant findings. Serological testing for *Echinococcus multilocularis* was negative.

**Figure 3.**
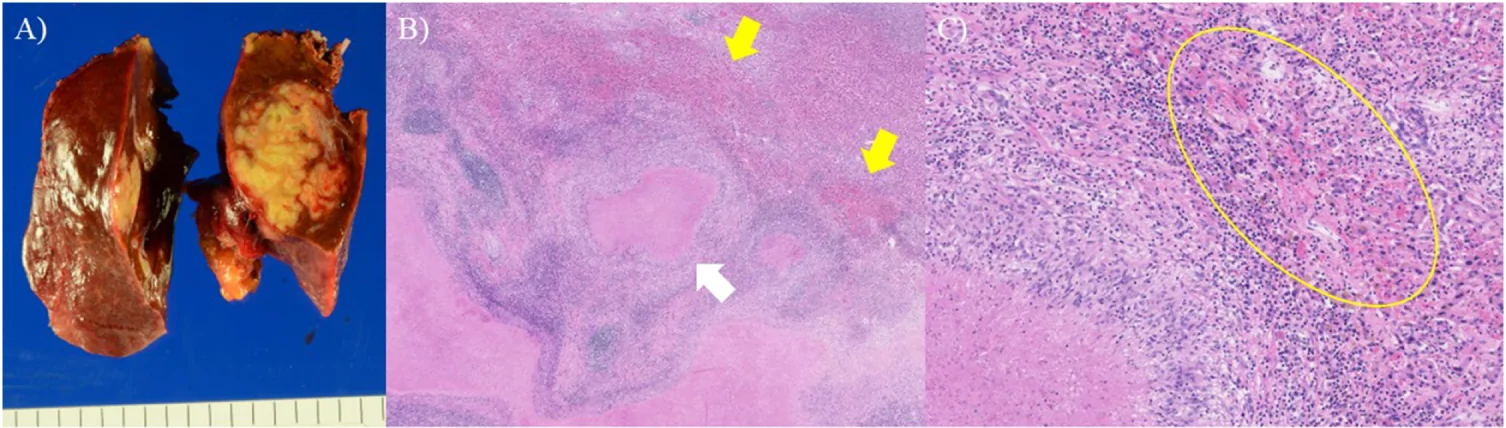
Gross and histopathological findings: (A) A relatively well-demarcated 40 mm mass composed of clustered small nodules. (B) A large granuloma with prominent eosinophilic infiltration (yellow arrows) and central coagulative necrosis (white arrow) (low-power field). (C) A cluster of eosinophils (high-power field).

Therefore, parasitic infection-associated HEG was considered unlikely, and the lesion was diagnosed as idiopathic HEG in the absence of peripheral eosinophilia. The patient has remained recurrence-free for more than 12 months after surgery.

## Discussion

EG is defined by characteristic histopathological findings associated with type I hypersensitivity; however, these features are not disease-specific and can appear in conditions such as necrotizing vasculitis, sarcoidosis, and Langerhans cell histiocytosis^[^1^]^. EG may also arise from drug-induced hypersensitivity or parasitic infections such as fascioliasis, ascariasis, clonorchiasis, schistosomiasis, and anisakiasis^[^3–10^]^. Because parasites commonly reach the liver via the portal circulation, HEG often represents a localized allergic reaction to migrating helminths^[^1^]^. However, in our case, neither prior medical history nor clinical findings suggested a drug allergy or parasitic exposure as the cause. Additionally, the absence of peripheral eosinophilia in preoperative laboratory tests further complicated the inclusion of HEG in the differential diagnosis.

Our previous review was based on a PubMed search (conducted in December 2025) using the term “hepatic eosinophilic granuloma.” Of the 52 articles identified, we analyzed seven detailed reports to identify diagnostic trends (Table 2)^[^1,5,8–12^]^. Serological evaluations were performed in six of these reports. Among the cases with clearly described diagnostic methods, nine were confirmed by surgical resection and five by biopsy. Most critically, HEG was suspected preoperatively in only one of the nine surgically diagnosed cases^[^1^]^. Manor *et al* reported a case of HEG without preoperative peripheral blood eosinophilia; however, the intralesional parasite was identified histopathologically, confirming parasitic infection^[^8^]^. Based on previous reports, HEG has a favorable prognosis following surgical resection but is rarely diagnosed preoperatively and is often identified only after histopathological examination of the resected specimen. Therefore, accurate non-invasive diagnostic methods are needed to guide appropriate surgical management.

**Table 2:** Diagnostic approaches in reported cases of HEG.

Published	Age, sex	Preoperative eosinophilia	Preoperative tumor marker	Preoperative diagnosis	Method of definitive diagnosis	Final diagnosis	Recurrence
Saito *et al* 2008^[^5^]^	68, M	71%	Negative	Pancreatic cancer with liver metastasis	Liver biopsy	HEG caused by VLM	NA
Hassan *et al* 2013^[^9^]^	17–56, M3, F5	All 8 cases (26%–72%)	NA	NA	Three biopsies and five partial hepatectomies	Eosinophilic granulomatous liver abscesses	No recurrence
Yamamoto *et al* 2015^[^1^]^	43, F	10%	Negative	Inflammatory pseudotumor or VLM-related HEG	Laparoscopic S6 subsegmentectomy	HEG due to unknown cause	No recurrence
Manor *et al* 2021^[^8^]^	59, M	No	NA	VLM (*C. hepatica*/*Ascaris*)	S7 subsegmentectomy	Asymptomatic hepatic capillariasis	NA
Yamada *et al* 2023^[^10^]^	71, M	NA	NA	Liver tumor (S6)	Laparoscopic S6 partial hepatectomy	Hepatic anisakidosis	NA
Amiel *et al* 2024^[^12^]^	23, F	NA	Negative	Liver abscess (S4)	Laparoscopic biopsy	Crohn’s disease	No recurrence
Dai *et al* 2026^[^11^]^	53, M	NA	NA	Liver metastases (Unknown primary)	US-guided liver biopsy	HEG	NA
Our case 2026	67, F	No	Negative	Inflammatory pseudotumor or ICC	Laparoscopic S3 subsegmentectomy	HEG due to unknown cause	No recurrence

M, male; F, female; NA, not available; S3/S4/S6/S7, segment3/4/6/7; VLM, visceral larval migrans

**Table 1:** Laboratory data on admission.

Complete blood count		Biochemistry		Coagulation tests	
White blood cells	5680/μL	Total bilirubin	0.5 mg/dL	Prothrombin time activity	101%
Neutrophils	60.8%	Direct bilirubin	<0.1 mg/dL	Prothrombin time–international normalized ratio	0.99
Lymphocytes	24.6%	Aspartate aminotransferase	35 U/L	Activated partial thromboplastin time	27.6 sec.
Monocytes	6.7%	Alanine aminotransferase	47 U/L	D-dimer	0.5 μg/mL
Eosinophils	7.2%	Lactate dehydrogenase	182 U/L		
Basophils	0.7%	Alkaline phosphatase	53 U/L	Tumor markers	
Absolute eosinophil count	410/μL	γ-glutamyl transpeptidase	22 U/L	Alpha-fetoprotein	3.5 ng/mL
Red blood cells	4.87 × 10^6^/μL	Cholinesterase	340 U/L	Protein induced by vitamin K absence or antagonist-II	17 mAU/mL
Hemoglobin	14.0 g/dL	Amylase	79 U/L	Carcinoembryonic antigen	1.4 ng/mL
Hematocrit	42.4%	Total protein	7.7 g/dL	Carbohydrate antigen 19–9	9.0 U/mL
Platelets	226 × 10^3^/μL	Albumin	4.2 g/dL	S-pancreas-1 antigen	3.6 U/mL
		Blood urea nitrogen	10.0 mg/dL	Duke pancreatic monoclonal antigen type 2	< 25 U/mL
		Creatinine	0.47 mg/dL		
		Total cholesterol	205 mg/dL	Indocyanine green retention rate at 15 minutes	11.3%
Immunoglobulin G4	11 mg/dL	C-reactive protein	1.24 mg/dL	Indocyanine green plasma disappearance rate	0.141
Soluble interleukin-2 receptor	557 U/mL	Sodium	140 mmol/L	Hepatitis B surface antigen	Negative
Antinuclear antibody	<1:40	Potassium	4.0 mmol/L	Hepatitis B surface antibody	Negative
		Chloride	104 mmol/L	Hepatitis C virus antibody	Negative
		Blood sugar	95 mg/dL		
		Hemoglobin A1c	6.0%		

A treatment strategy is often selected based on preoperative findings. Surgical resection is accepted for diagnostic and therapeutic purposes. Hepatic granulomas caused by infection are primarily treated with appropriate antimicrobial, antifungal, antiviral, or antiprotozoal medications, which represent the gold standard treatment^[^1^]^. Radiologically, HEG often demonstrates peripheral, faint, and reticular enhancement with poor central enhancement, which differs from the typical vascular patterns seen in hepatic malignancies on dynamic contrast-enhanced CT^[^5,7^]^. Hepatocellular carcinoma (HCC) usually presents with arterial hyperenhancement, followed by washout and a hepatobiliary-phase defect, whereas ICC commonly presents with mild arterial enhancement and progressively delayed enhancement due to fibrotic stroma. In addition, HEG may exhibit high peripheral signal intensity on diffusion-weighted imaging without marked central diffusion restriction, whereas HCC and ICC generally demonstrate more pronounced diffusion restriction. During the hepatobiliary phase, HEG may show minimal or heterogeneous uptake, whereas malignant tumors typically appear as clear defects. These imaging differences may aid in distinguishing HEG from HCC or ICC. Preoperative imaging does not sufficiently distinguish HEG from HCC, ICC, and metastatic liver tumors. Although percutaneous biopsy enables definitive diagnosis, if the lesion is predominantly necrotic, adequate sampling can be difficult, potentially resulting in a false-negative diagnosis, and there is a potential risk of needle-tract seeding. Therefore, minimally invasive surgical resection was selected as a diagnostic and therapeutic strategy.

Immunohistochemical staining did not reveal any significant findings. Ziehl–Neelsen, Gram, Grocott, and periodic acid–Schiff staining revealed no evidence of bacterial or fungal pathogens, including acid-fast bacteria or fungi. Interferon-gamma release assay (T-SPOT.TB) and screening tests for antiparasitic antibodies (SRL Inc., Tokyo, Japan) were performed, and the results were all negative. The patient had no history of parasitic infection or relevant exposure such as consuming raw freshwater fish, traveling overseas, or coming into contact with foxes. No pathological findings suggested parasitic infection, including clonorchiasis, and there was no evidence of bile stasis. Serological testing for echinococcosis yielded negative results. Thus, no specific infection or other underlying cause of HEG could be identified, and the lesion was ultimately diagnosed as an idiopathic HEG.

A definitive diagnosis of HEG requires either histopathological identification of parasitic organisms or positive serological testing; therefore, establishing a diagnosis remains challenging when a parasitic infection is not suspected, especially without peripheral blood eosinophilia. Accordingly, HEG should be considered in the differential diagnosis for hepatic lesions, even in patients without peripheral blood eosinophilia or an apparent risk of parasitic infection. In cases such as ours, where distinguishing HEG from hepatic malignancy is particularly difficult, surgical intervention for diagnostic and therapeutic purposes may be justified. The accumulation of additional cases is essential to further clarify the optimal diagnostic approach for this rare entity.

This report has some limitations. First, as this was a single-case report, the clinical features, diagnostic approach, and treatment outcomes cannot be easily generalized to other patients with HEG. Second, despite extensive pathological and serological evaluation, the pathogenesis of HEG remains unclear.

We report a case of HEG in the absence of identifiable parasitic infections or peripheral eosinophilia. HEG should be considered in the differential diagnosis of hepatic tumors.

## Data Availability

Not applicable.
